# Phase II Clinical Trial of Second Course of Stereotactic Body Radiotherapy for Spinal Metastases

**DOI:** 10.3390/cancers16122286

**Published:** 2024-06-20

**Authors:** Kei Ito, Yujiro Nakajima, Kentaro Taguchi, Hiroaki Ogawa, Makoto Saito, Keiko Nemoto Murofushi

**Affiliations:** 1 Division of Radiation Oncology, Department of Radiology, Tokyo Metropolitan Cancer and Infectious Diseases Center, Komagome Hospital, 3-18-22 Honkomagome, Bunkyo-ku, Tokyo 113-8677, Japan; 2 Department of Radiological Sciences, Komazawa University, 1-23-1 Komazawa, Setagaya-ku, Tokyo 154-8525, Japan; 3 Department of Radiation Oncology, Tohoku University Graduate School of Medicine, 1-1 Seiryo-machi, Aoba-ku, Sendai 980-8574, Japan; 4 Division of Clinical Research Support, Tokyo Metropolitan Cancer and Infectious Diseases Center, Komagome Hospital, 3-18-22 Honkomagome, Bunkyo-ku, Tokyo 113-8677, Japan

**Keywords:** adverse effect, prospective clinical trial, second course, stereotactic body radiotherapy, spinal metastases

## Abstract

**Simple Summary:**

This single-center, single-arm, phase II trial aimed to propose a safe and effective salvage spine SBRT regimen for patients with spinal metastases. The second SBRT dose consisted of 30 Gy delivered in five fractions with specific dose constraints for the spinal cord and nerve plexuses. Among the enrolled patients, 12 received the second SBRT at the same spinal level, while 8 received it at an adjacent level. No instances of radiation myelopathy or local failure were observed during the follow-up period. However, grade 3 late adverse effects (including lumbosacral plexopathy and vertebral compression fractures) were observed in 25% of patients throughout the entire follow-up period, suggesting that the second SBRT poses a risk of toxicity.

**Abstract:**

Purpose: The optimal method for the second course of stereotactic body radiotherapy (SBRT) for spinal metastases remains poorly established. This single-center, single-arm, phase II trial was conducted to propose a safe and effective salvage spine SBRT. Methods: The patients initially treated with SBRT for spine-targeted protocol treatment, or for areas adjacent to the spine, were enrolled. The second SBRT dose was 30 Gy delivered in five fractions; the spinal cord dose constraint was 15.5 Gy at the maximum point dose. The brachial or lumbosacral plexuses were dose-constrained to <30 Gy if the boundary between the nerves and tumors was detected. The primary endpoint was dose-limiting toxicity (DLT) (grade ≥ 3 severe radiation-related toxicity) within a year after the second SBRT. Results: The second SBRT was administered to the same spinal level in 12 patients and to an adjacent spinal level in 8 patients. SBRT2 was performed for 14 painful lesions, 10 MESCC, and 6 oligometastases, with some lesions having multiple indications. The median interval between SBRT sessions was 21 months (range: 6–51 months). The median follow-up duration was 14 months. No radiation myelopathy or local failure was reported during the follow-up period. DLT was confirmed in two patients (10%) within a year, both of whom developed grade 3 lumbosacral plexopathy. These two patients received SBRT twice to the S1–2 and S1–5 vertebrae, respectively, and both experienced paralysis of the tibialis anterior muscle (L5 level). Grade 3 late adverse effects (including lumbosacral plexopathy and vertebral compression fracture) were observed in 25% of the patients throughout the entire follow-up period. Conclusions: The second spine SBRT achieved good local control without causing myelopathy. However, one-quarter of the patients experienced grade 3 late adverse effects, suggesting that the treatment protocol carries a risk of toxicity.

## 1. Introduction

The bone is the third most common site of metastasis, following the liver and lungs [1]. According to data from large-scale clinical trials, 30–40% of painful bone metastases occur in the spine [2,3,4]. Currently, conventional external beam radiotherapy (EBRT) is the gold standard treatment for spinal metastases [5,6].

Stereotactic body radiotherapy (SBRT) is a high-precision technique that delivers a high ablative biological dose in a few fractions while sparing the adjacent at-risk organs. The indications for spine SBRT include painful lesions, metastatic epidural spinal cord compression (MESCC), and oligometastases. A recent phase III trial, SC.24, demonstrated that SBRT could afford substantially superior pain relief when compared with that which was elicited by conventional EBRT [7]. Consequently, SBRT has become one of the standard treatments for painful spinal metastases. Regarding MESCC, two phase II trials of postoperative SBRT have consistently shown a one-year local failure (LF) rate of approximately 10%, accompanied by a high probability of maintaining or improving ambulatory function [8,9]. Considering oligometastases, a randomized phase II study, the SABR-COMET trial, reported the survival benefit of SBRT when compared with that of standard care [10]. These promising outcomes of spine SBRT have resulted in its increased implementation in daily clinical practice.

Spine SBRT has achieved an impressive tumor control rate of 80–90% [11]; nevertheless, over 10% of patients experience LF. For patients with LF, after the initial SBRT course (SBRT1), a second SBRT course (SBRT2) can be considered [12]. Nonetheless, the risk of plexopathy was recently reported to be clinically relevant, occurring in more than 20% of patients treated with salvage SBRT2 for spinal metastases [13]. To mitigate the risk of plexopathy even with the use of SBRT2, we hypothesized that reducing the dose to the brachial or lumbosacral plexuses would be beneficial, as the aforementioned study did not apply dose constraints to those nerves [13]. To establish a safe re-irradiation protocol for spinal metastases, we conducted the current clinical trial of SBRT2 with dose constraints on the brachial or lumbosacral plexuses.

## 2. Materials and Methods

### 2.1. Patients

The inclusion criteria were as follows: patients (1) who were pathologically or clinically diagnosed with spinal metastases; (2) with a history of SBRT to the same spine or the adjacent spine level; (3) who exhibited LF in the same spinal segments previously treated with SBRT or developed new lesions in the adjacent spine as shown on magnetic resonance imaging (MRI); (4) whose histological examination showed no radiation-sensitive primary tumors such as malignant lymphomas, myelomas, or seminomas; (5) whose interval from the SBRT1 to registration was more than 6 months; (6) who can remain in a supine position for 1 h; (7) who were aged ≥ 20 years; and (8) who had a predicted overall survival (OS) of >6 months.

The present study performed spine SBRT for painful lesions, MESCC, and oligometastases. The indications were the same for both SBRT1 and SBRT2. For patients with MESCC, separation and fixation surgery prior to SBRT was conducted if tolerable. No upper limit was set for the number of spinal segments targeted.

### 2.2. Study Design

This open-label, single-center, single-arm, prospective phase II study evaluated the clinical outcomes of SBRT2 in patients with spinal metastases. The primary endpoint was dose-limiting toxicity (DLT) within one year of SBRT2 completion. DLTs were defined as grade 3 gastritis, enterocolitis, myelitis, brachial plexopathy/plexopathy, grade 4 radiation dermatitis, pharyngeal/laryngeal mucositis, esophagitis, tracheitis, or acute kidney injury. Vertebral compression fractures (VCFs) were not included as DLTs. The secondary endpoints were OS, pain response, LF, and other adverse effects (AEs).

The study was approved by our institutional ethical review board (approval number: 2675), and written informed consent was obtained from all patients. This study was registered in the University Hospital Medical Information Network Clinical Trials Registry (UMIN000043319).

### 2.3. Stereotactic Body Radiotherapy

SBRT for spinal metastases was conducted with palliative intent for painful lesions, to improve neurological function for MESCC, or with curative intent for oligometastases [14].

The methodologies used were basically common to SBRT1 and SBRT2. Patients were positioned supine and secured using a thermoplastic head-and-shoulder mask (CIVCO Medical Solutions, Kalona, IA, USA), along with a head-and-shoulder vacuum cushion (Vac-Lok cushion; CIVCO Medical Solutions, Kalona, IA, USA) for individuals with cervical or upper-thoracic (typically to Th3) spinal lesions. For those with midthoracic or lower spinal lesions, a full-body Vac-Lok cushion was utilized. Planning computed tomography (CT) scans were conducted with a slice thickness of 1 mm. Subsequently, all patients underwent MRI to delineate the tumor, spinal cord, or cauda equina. Alternatively, CT myelography was performed for patients who were ineligible for an MRI due to the presence of a pacemaker and other contraindications or metal artifacts hindering spinal cord detection.

The clinical target volume (CTV) included the gross tumor volume as well as the immediately adjacent bony anatomic compartments, which might harbor microscopic disease, following the contouring guidelines for spine SBRT [15,16,17]. The CTV for SBRT2 did not necessarily encompass the entire area of the tumor treated by SBRT1. A 2 mm margin was added to the CTV to establish the planning target volume (PTV). The spinal cord and cauda equina were contoured using a non-enhanced MRI. A 1.5 mm margin was added to the spinal cord and defined as the planning organ-at-risk volume of the cord (PRV_cord_). The thecal sac for the cauda equine was delineated without margins. Other at-risk organs, including the brachial and lumbosacral plexuses, were delineated based on the images acquired using simulation CT. However, the nerves were not contoured if gross tumor and nerve root boundaries were not detected. The PRV margin was not added to the brachial or lumbosacral plexuses.

**Figure 1:**
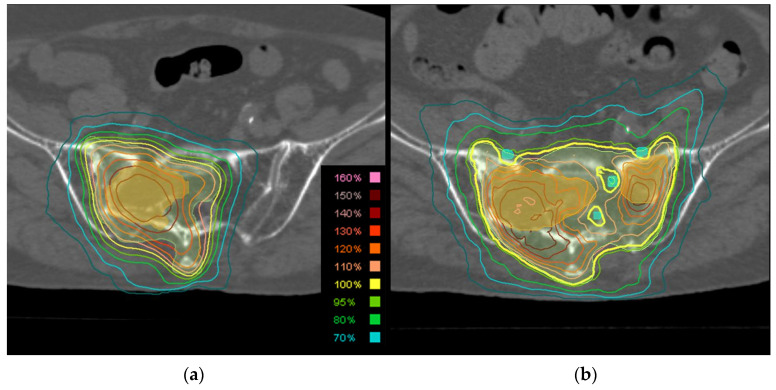
Imaging findings in an 85-year-old male patient with sacral spinal metastases from prostate cancer. (**a**) Axial computed tomography images with contouring (orange = gross tumor volume and light green = planning target volume) and dose distribution of initial stereotactic body radiotherapy (SBRT) of 24 Gy in two fractions to the S2–5 level. (**b**) Axial computed tomography images during the second SBRT course, when 30 Gy in five fractions were administered to the S1–5 level. Although the sacral nerves were dose-constrained to <30 Gy, it failed to circumvent the right S1 and S2 nerve roots due to tumor invasion, resulting in almost complete paralysis of the right tibialis anterior muscle after 4 months.

The prescribed SBRT2 dose was 30 Gy delivered in five fractions on the basis of a previous retrospective case series [13,18]. The objective was to guarantee that 95% of the PTV received the prescribed dose, while ensuring that the normal tissues adhered to dose constraints. Furthermore, limits were placed on 2% of the PTV to receive less than 160% of the prescribed dose. Dose constraints were established for the PRV_cord_ and thecal sac, ensuring that the maximum point dose did not exceed 15.5 Gy in five fractions [19], with the maximum point dose defined as ≤0.035 cc [20]. The dose constraint for the brachial or lumbosacral plexuses was <30 Gy (Figure 1). The prescribed doses and dose constraints are listed in Supplementary Materials (Table S1).

### 2.4. Evaluation and Statistical Analyses

Follow-up assessments and MRI scans were conducted at 3, 6, 9, and 12 months following the implementation of the treatment regimen. AEs were assessed in accordance with version 5 of the National Cancer Institute’s Common Terminology Criteria for Adverse Events [21]. Acute AEs were those occurring within 90 days of initiating the treatment protocol, while late AEs manifested 90 days after treatment initiation. Radiologists diagnosed radiation myelopathy (RM) based on the T2-weighted and enhanced T1-weighted MRI scans [22]. RM severity was graded using the Radiation Therapy Oncology Group Radiation Morbidity Scoring System and the European Organization for Research and Treatment of Cancer [23]. Plexopathy was diagnosed by the presence of focal neurological deficits in the irradiated area without LF or RM. VCFs were defined as the appearance of new VCFs or the progression of existing ones in vertebral bodies which were irradiated, as evidenced by the MRI or CT scans. OS was calculated from the registration date to the most recent follow-up date or death from any cause. Pain response was evaluated following the International Consensus Criteria [24]. The pain response was determined based on the highest score reported in the previous 3 days on a numerical rating scale of 0–10 and changes in opioid analgesic dosage [24]. LF was defined as tumor progression or the appearance of a new tumor within the epidural space, as detected by MRI (or CT in certain situations), following the recommendations of the Spine Response Assessment in Neuro-Oncology group [25].

Twenty patients were selected to assess the minimum safety. Assuming the occurrence of one, two, and three DLTs, the 90% confidence intervals for the occurrence rates were 0.003–0.216, 0.018–0.283, and 0.042–0.3444, respectively. We opted to limit the occurrence of DLTs to a maximum of 30%, with the treatment protocol considered safe if DLT only occurred in ≤2 patients (upper confidence interval < 0.3).

OS was estimated using the Kaplan–Meier method. Death without tumor recurrence was considered a competing risk factor. LF was estimated using the cumulative incidence function adjusted for the competing risk of death. All statistical analyses were performed using the EZR software version 1.54 [26].

## 3. Results

### 3.1. Patient and Tumor Characteristics

Overall, 20 patients with 20 spinal lesions were enrolled between April 2021 and March 2022. Table 1 summarizes the patient and tumor characteristics. Ten lesions (50%) were radioresistant, including thyroid cancer (20%), soft tissue sarcoma (20%), and bone sarcoma (10%). SBRT2 was performed for 14 painful lesions, 10 MESCC, and 6 oligometastases, with some lesions having multiple indications. SBRT2 was delivered to the same spinal level as SBRT1 in 12 lesions and to the adjacent spinal levels to SBRT1 in 8 lesions. The prescribed SBRT1 dose was 20 Gy, delivered as a single fraction in five patients, and 24 Gy, delivered in two fractions in 15 patients. Dose constraints at SBRT1 were set for the thecal sac and PRV_cord_ such that the maximum point dose was <12.4 or 14.0 Gy in a single fraction, <17.0 Gy in two fractions for radiation-naive patients, and <12.2 Gy in two fractions for irradiated patients. The mean and median intervals from SBRT1 to SBRT2 were 22.4 and 21 months (range: 6–51 months), respectively. Prior to SBRT1, five lesions (25%) were initially irradiated with conventional EBRT, meaning that these five lesions were subjected to a total of three sessions of radiotherapy. The mean interval from conventional EBRT to SBRT1 was 13.8 months (range: 8–18 months). At the time of SBRT2, ten lesions (50%) with spinal cord compression with a Bilsky grade ≥ 1c were detected [27]. Of these ten lesions, only one had associated motor paralysis, with a manual muscle test (MMT) score of four. The median SBRT2 dose to 95% of the PTV was 27.9 Gy (range: 18.7–30.0 Gy).

### 3.2. Clinical Outcomes

The median follow-up period following SBRT2 was 14 months (range: 2–31 months). For the entire cohort, the one-year OS rate and median survival time were 65% and 16.3 months, respectively (Supplementary Materials, Figure S1). LF was not confirmed during the follow-up period. Considering 14 painful lesions (≥2 points on a 0–10 scale), the overall pain response rate (complete plus partial response) at 1, 3, 6, 9, and 12 months was 62% (8/13 lesions), 60% (6/10 lesions), 38% (3/8), 50% (4/8), and 67% (4/6), respectively (Table 2).

Table 3 summarizes the incidence of maximum AEs observed throughout the follow-up period. In the acute phase, grade 3 AEs were observed in one patient. The patient received SBRT to the C1–3 spine twice, with an interval of 24 months. Oral mucositis developed 2 weeks after SBRT2 and resolved spontaneously within 1 week (not qualifying as a DLT).

In the late phase, all grades of RM, plexopathy, and VCFs were observed in 0, 4 (20%), and 5 (25%) patients, respectively. For patients with lesions at the C4–Th1 and L1–S2 levels, which control the motor and sensory functions of the limbs, 36% (4/11) of patients developed plexopathy. DLT within a year of the primary endpoint was confirmed in two (10%) patients. However, DLT during the entire follow-up period was confirmed in three (15%) patients, all of whom experienced grade 3 lumbosacral plexopathy. Grade 3 late AEs, such as plexopathy and VCFs, were observed in 25% of the patients throughout the entire follow-up period. (Detailed information is shown in the next section and Table 4). Other grade ≥3 AEs were not encountered.

### 3.3. Detailed Information on Patients with Grade 3 Late AEs

Table 4 presents detailed information regarding three patients with grade 3 plexopathy. All three patients received SBRT2 to the sacrum and experienced severe paralysis of the tibialis anterior muscles. These patients walked using the aid of a cane owing to foot drop gait. Considering the treatment plan for patient 1, dose constraints for the sacral nerves were met; however, in the remaining two patients’ plans, the nerves were not preserved owing to tumor infiltration in the nerve roots. Among the 11 treatment plans, including those delivered to the C4–Th1 and L1–S2 levels, the nerves were preserved in 5 plans (grade 3 plexopathy occurrence: 20%). Meanwhile, the nerves could not be avoided in the remaining six plans (grade 3 plexopathy occurrence: 33%).

Grade 3 VCFs were confirmed in two patients during follow-up. One patient was a 58-year-old male with prostate cancer. SBRT1 24 Gy was administered in two fractions to the L3 vertebra 41 months before SBRT2, and SBRT2 was later performed on the same spinal segment. The spinal instability neoplastic score was three (location of mobile spine and mixed bone lesion). Progression of the existing VCF was confirmed in the L3 vertebra 22 months after SBRT2. The patient underwent fixation surgery for severe pain due to instability. The other patient was a 74-year-old male with esophageal cancer. SBRT1 24 Gy was administered in two fractions to the L4–5 vertebrae 16 months before SBRT2, and SBRT2 was later administered to the same spinal segment. The spinal instability neoplastic score of L3 was two (location of mobile spine). The new VCF was confirmed in L3 4 months after SBRT2. The patient underwent surgery for compression due to a spinal canal stenosis, which led to paralysis of the left tibialis anterior muscles (L5 level, MMT 4), extensor hallucis longus muscle (L5 level, MMT 2), and flexor hallucis longus muscle (L5–S1 level, MMT 4). Following the decompression surgery, the paralysis of the extensor hallucis longus muscle improved, prompting its reclassification as a VCF rather than plexopathy.

## 4. Discussion

Herein, we conducted the first prospective trial to propose a safe SBRT2 protocol for treating spinal metastases. We employed SBRT to spare not only the spinal cord but also the brachial and lumbosacral nerve roots. The treatment protocol involved the delivery of 30 Gy of radiation in five fractions, eliciting excellent local control and the absence of RM, severe laryngeal mucositis, and esophagitis. However, three patients experienced grade 3 plexopathy, and two patients experienced grade 3 VCFs throughout the entire follow-up period.

Spine SBRT has three objectives as follows: pain control for patients with painful lesions, prevention of or improvement in MESCC-induced neurological deficits, and extended OS for individuals with oligometastases [14]. Several studies have demonstrated promising outcomes across these objectives, leading to an increased application of SBRT in daily clinical practice. With the increased application of SBRT, the incidence of LF also increases. However, the optimal strategies for salvage re-irradiation after SBRT have yet to be established. Accordingly, the objective of the current clinical trial was to develop a safe and effective SBRT2 method.

Herein, we discussed the optimal dose–fraction schedules for SBRT2 based on published studies. Two retrospective studies of SBRT2 in spinal metastases have reported [13,18] delivering 30 Gy in four fractions (median dose) [18] and 30 or 35 Gy in five fractions [13]. The latter revealed the absence of LF within 1 year or RM in 19 lesions [13]. Additionally, another study has reported a one-year LF rate of 19% and no occurrence of RM in 56 lesions [18]. The present trial, using an SBRT2 dose of 30 Gy delivered in five fractions, also detected no LF or RM in 20 lesions. Therefore, SBRT2 at 30–35 Gy in four to five fractions could be applied in daily clinical practice from the viewpoint of local control and safety of the spinal cord. When performing SBRT2, the prescribed dose and dose constraints used in the present trial would be informative.

SBRT for de novo spinal metastases and re-irradiation SBRT after conventional EBRT has been reported to rarely cause plexopathy [11,28]. Conversely, more than one course of SBRT to the same site has the risk of causing plexopathy [13,29]. A retrospective study about SBRT2 confirmed a high rate of grade 3 plexopathy in 16% (3/19) of the entire cohort and 27% (3/11) of lesions at the C4–Th1 and L1–S2 levels [13]. Hence, the present trial adopted the SBRT2 method that spares the nerve roots if GTV and the nerve roots were separated. Herein, we reported grade 3 plexopathy in 15% (3/20) of the whole cohort and 27% (3/11) of the lesions at the C4–Th1 and L1–S2 levels, demonstrating that the method had no significant advantage. Spinal metastases frequently occur in the posterior region of the vertebral body and pedicle [30]; therefore, an SBRT plan that irradiates the gross tumor with a sufficient dose cannot avoid the nerve roots in many cases. Additionally, in cases of mass-type metastases, it is impossible to detect the nerve roots separately from the tumor. Therefore, preventing plexopathy during SBRT2 remains an unresolved challenge. If salvage irradiation is not administered, recurrent tumors invade the nerve roots and cause plexopathy. Appropriate therapeutic strategies should be determined based on the advantages and disadvantages of SBRT2. These results will be useful for appropriately guiding treatment decisions.

Tolerance doses for the brachial and lumbosacral plexuses have yet to be established. The American Association of Physicists in Medicine (AAPM) Task Group (TG)-101 report recommends 16 Gy in one fraction and 32 Gy in five fractions as the maximum dose [20] in SBRT for radiating naïve lesions. Even in the absence of specific dose constraints for the aforementioned nerves, the overall peripheral nervous injury rate was only 2.5% in a retrospective case series of 557 high-dose, single-fraction SBRT sessions performed for de novo spinal metastases [31]. It should be noted that re-irradiation SBRT without dose constraints for the brachial and lumbosacral plexuses after conventional EBRT caused plexopathy in only 1.5% (2/133) of lesions [32]. In contrast, the present study revealed a high probability of repeated SBRT-inducing severe plexopathy. The tolerance dose of the brachial and lumbosacral plexuses is presumed to be higher than that which has been recommended by the AAPM TG [20] but lower than the cumulative dose of SBRT administered twice. In particular, the CTV is extensive in sacral SBRT [17], with the sacral nerves extensively irradiated, potentially leading to damage. To mitigate the risk of plexopathy in SBRT2, effective countermeasures may include the shrinkage of the CTV, a reduction in the prescribed dose, or the prevention of an increase in the central dose. Future studies should undertake dosimetric analyses to clarify the tolerance dose of nerve roots.

The occurrence rate of grade 3 VCFs caused by SBRT1 varies. In the SC.24 phase III trial, which compared the palliative effect between conventional EBRT and SBRT for painful spinal metastases, SBRT caused grade 3 VCF in 1% of the patients (1/110 patients) [7]. A retrospective case series at our institution found grade 3 VCFs in 1.9% of all the irradiated spinal segments (15/779 segments) [33]. On the other hand, several retrospective studies have reported a high grade 3 VCF occurrence rate of 15% (15/100 segments) and 21% (15/72 segments) [34,35]. The present study confirmed SBRT2-induced grade 3 VCFs in 10% of the patients. This occurrence rate is uncertain due to the small sample size and number of events. In addition, it is unclear whether the 10% occurrence rate is higher than that of SBRT1. More data are required to clarify the risk of severe VCFs induced by SBRT2.

The limitations of the current study need to be addressed. First, the sample size and the number of plexopathy events were markedly limited to yield conclusive results. Second, the follow-up duration was relatively short (median: 14 months; range: 2–31 months). Assuming that radiation-related neurotoxicity occurs in the late phase, the rate of occurrence observed in our study may have been underestimated. Therefore, large-scale studies with long-term follow-ups are warranted. Third, VCF was not included in the DLT. We did not consider it relevant to the decision-making process because most SBRT-induced VCFs are painless [7,33]. However, the rare occurrence of a severe VCF (e.g., grade 3: intensely painful, requiring hospitalization or invasive interventions such as percutaneous cement injection or surgery) had a strong impact on the patient’s quality of life and influenced the treatment decision-making process.

## 5. Conclusions

We conducted the first prospective clinical trial regarding a second course of salvage SBRT, delivered at a dose of 30 Gy in five fractions to patients previously treated with spine SBRT. This approach achieved excellent local control without RM, severe laryngeal mucositis, and esophagitis. On the other hand, radiation-induced plexopathy was observed despite our efforts to minimize this AE. Grade 3 late AEs were observed in 25% of patients throughout the entire follow-up period, suggesting that the treatment protocol carries a risk of toxicity.

## Figures and Tables

**Table 1:** Patient characteristics.

Characteristic		20 Patients
Sex	Male/Female	11/9
Age, years	Median (range)	66 (36–85)
Lesion histology	Soft tissue sarcoma	4
	Thyroid	4
	Prostate	3
	Bladder	2
	Bone sarcoma	2
	Breast	2
	Lung	2
	Esophagus	1
Indications for SBRT2 ^1^	Painful lesions	14
	MESCC	10
	Oligometastases	6
Levels treated ^2^	Cervical/Thoracic/Lumbar/Sacral	1/11/6/4
Number of spinal levels	1/2/3/4–5	8/4/6/2
SINS	Stable/Potentially unstable/Unstable	4/11/5
Bilsky grade	0–1b/1c/2/3	10/4/6/0

SINS, spinal instability neoplastic score. ^1^ Some patients had multiple indications for irradiation. ^2^ Two patients had lesions across the thoracic and lumbar areas.

**Table 2:** Pain response over time.

	All Lesions, Number (%)				
	1 Month	3 Months	6 Months	9 Months	12 Months
Responders	8/13 (62)	6/10 (60)	3/8 (38)	4/8 (50)	4/6 (67)
CR + PR	4 + 4	2 + 4	1 + 2	1 + 3	2 + 2
Non-responders	5/13 (38)	4/10 (40)	5/8 (63)	4/8 (50)	2/6 (33)
PP + IR	1 + 4	1 + 3	2 + 3	2 + 2	1 + 1

CR, complete response; IR, indeterminate response; PP, pain progression; PR, partial response.

**Table 3:** Adverse effects of the second stereotactic body radiotherapy course.

	Grade 1	Grade 2	Grade 3	Grade 4–5
Acute phase	4 (20%)	0	1 (5%)	0
Oral mucositis	0	0	1	0
Nausea	1	0	0	0
Esophagitis	2	0	0	0
Edema limbs	1	0	0	0
Late phase	3 (15%)	2 (10%)	5 (25%)	0
Plexopathy	0	1	3	0
Dysphagia	1	0	0	0
Fracture	2	1	2	0

**Table 4:** Patients with grade 3 plexopathy.

No.	Age (Years)/ Sex	Type of Cancer	Previous Radiotherapy (Time)	Site of SBRT2	Symptoms of Plexopathy	Time to Onset (Months)
1	77/Female	Thyroid cancer	SBRT of 24 Gy in 2 fractions at the L5 vertebral body (11 months earlier)	S1 right sacral wing	Paralysis of the right tibialis anterior muscles (L5 level, MMT 2)	18
2	55/Female	Soft tissue sarcoma	SBRT of 20 Gy in a single fraction at the S1–2 vertebral body and left sacral wing (10 months earlier)	S1–2 vertebral body and bilateral sacral wing	Paralysis of the left tibialis anterior muscles (L5 level, MMT 2)	3
3	85/Male	Prostate cancer	8 Gy in a single fraction at the S1–5 level (37 months earlier) SBRT of 24 Gy in 2 fractions at the S2–5 vertebral body and right sacral wing (24 months earlier)	S1–5 vertebral body and bilateral sacral wing	Paralysis of the right tibialis anterior muscles (L5 level, MMT 1)	4

MMT: Manual muscle test, SBRT: Stereotactic body radiotherapy, SBRT2: Second SBRT course.

## Data Availability

The datasets used and/or analyzed during this study are available from the corresponding author on reasonable request.

## Supplementary Materials

The following supporting information can be downloaded at: https://www.mdpi.com/article/10.3390/cancers16122286/s1, Figure S1: Kaplan–Meier curve of overall survival after registration; Table S1: Prescribed dose and dose constraints.
