# Supplementary material for: Phase II Clinical Trial of Second Course of Stereotactic Body Radiotherapy for Spinal Metastases
Source: Cancers (Basel). 2024 Jun 20;16(12):2286. doi: 10.3390/cancers16122286 (PMC11201663; doi:10.3390/cancers16122286)
Supplement: Supplementary file 1 [file cancers-16-02286-s001.zip › cancers-2948233-supplementary.pdf]

Table S1. Prescribed dose and dose constraints.

Organs (PRVs)	Dose per five fractions
PTV	$D_{95\%}$ ≤ 30.0 Gy (100% PD) and
	$D_{2\%}$ ≤ 48.0 Gy (160% PD)
Brain	$D_{0.035\ cc}$ ≤ 30.0 Gy
Brain stem (+1.5-mm margin)	$D_{0.035\ cc}$ ≤ 15.5 Gy
Spinal cord (+1.5-mm margin)	$D_{0.035\ cc}$ ≤ 15.5 Gy
Cauda equina (thecal sac)	$D_{0.035\ cc}$ ≤ 15.5 Gy
Brachial or lumbosacral plexuses	$D_{0.035\ cc}$ ≤ 30.0 Gy
Pharynx	$D_{0.035\ cc}$ ≤ 29.0 Gy
Trachea/Bronchi	$D_{0.035\ cc}$ ≤ 29.0 Gy
Esophagus	$D_{2.5\ cc}$ ≤ 25.0 Gy and
	$D_{0.035\ cc}$ ≤ 29.0 Gy
Heart	$D_{15\ cc}$ < 20.0 Gy
Kidney (bilateral)	$D_{200\ cc}$ < 11.0 Gy
Stomach, small intestine, and colon (bowel bag)	$D_{0.035\ cc}$ ≤ 29.0 Gy
Skin (thickness of 3 mm)	$D_{1\ cc}$ ≤ 29.0 Gy

$D_{X\%/Y\ cc}$ Dose irradiated to the X%/Y cc, PD Prescribed dose, PRV Planning organ-at-risk volume, PTV Planning target volume

Figure S1. Kaplan–Meier curve of overall survival after registration

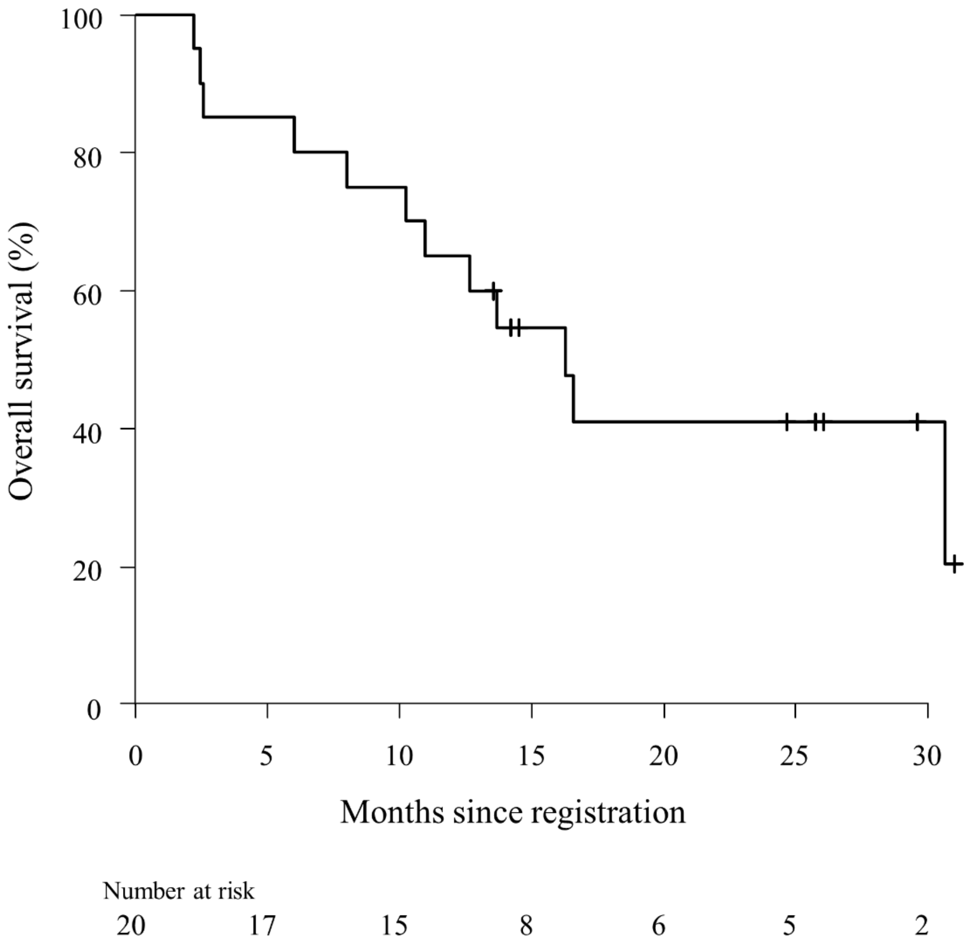

The 12-month survival rate and median survival are 65% and 16.3 months, respectively.
